# Dental Societies

**Published:** 1884-01

**Authors:** 


### DENTAL SOCIETIES.

That the wonderful advance of modern dental science is in a great measure due to the organization of dental societies throughout the world, no one will dispute. There can be but little professional progress without organized effort; the growth of dentistry as a distinct profession dates from the institution of dental societies, and the standing of the practising dentists in any locality may be accurately gauged by a perusal of the proceedings of their local society. The time is within the memory of many now in active life, when dentists locked their laboratories against the prying eyes of their neighbors, and closely guarded the information which was the result of their experience and study. That day of exclusiveness and unprofessional jealousy has happily passed away, and now every one worthy the name of a professional man hastens to give to his brethren the benefit of any discoveries which he may make, well knowing that it is only by giving freely that he can as freely receive, and that everything which tends to elevate his calling raises himself with it.

Dental societies are supposed to be instituted for the spread of professional information. Their mission is general, and the results of their sessions, if they be true to their ostensible object, belong to the profession at large. Any locking up of the papers and discussions, any selfish retention of them for ulterior purposes, is a violation of that broad code of ethics which demands that general professional information shall be made accessible to any one who seeks it. The dental journals are the recognized medium of communication for the diffusion of this very knowledge, and any society, therefore, that forbids the publication of its proceedings in respectable journals, is in spirit returning to the old narrow policy of jealously guarding the avenues of learning, lest haply some neighbor should gain a little surreptitious knowledge. If a valuable paper be presented, it belongs of right, not to the little favored clique who listened to its reading; not to the comparatively small body of men that makes up the membership of the society before which the essayist may read it, but to the profession at large. Science knows no boundary lines, no dividing walls, no society rules, no narrow personalism, but it is as broad as the world, as wide as the universe. We live in an age that cannot await the slow process of the publication of occasional volumes of transactions. We are making such rapid progress that any professional information demands instant spread, that it may assist those who are making investigations in the same field, and if it be delayed for but a short time, the world has passed the point when it would be valuable, and it becomes stale, flat, and unprofitable. We are nearing the point when a weekly dental journal will be demanded. General medicine has long since passed that period, and daily medical journals have been established. This is not an era of annuals, and labored and elaborate tomes have given place to brief and pithy daily, weekly, or at most monthly paragraphs.

The society that locks up its valuable information until in its own good time it shall choose to publish a volume of transactions is robbing its neighbors, and refusing that free interchange of ideas which marks every progressive association, and in reality belongs to the old era of closed laboratories, secret formulas, and exclusiveness. Volumes of transactions are not published for the world, but are the property of members. As a permanent record of what has been accomplished they are valuable, but for the purpose of spreading professional information they are comparatively useless.

We have been led to these remarks through the difficulty which we have experienced in obtaining reports of societies whose discussions were of value to the profession. When we have employed a reporter for the purpose of giving to our readers an account of the doings of some professional body, his requests for information and facilities for obtaining a report have been met by showing him some eighteenth century by-law, which forbids the publication of the proceedings until they have first appeared in the volume of transactions that appears at intervals of one, two, or three years. To our apprehension this is but a poor way of compassing the ends for which such organizations are established, and we cannot but urge that all dental societies become as liberal collectively, as most of their members are individually. If dental journals are to fulfill their true mission and be of the greatest benefit to the profession, an active co-operation is demanded by them of all those who love their calling. If information is to be given by them it must first be received, for editors are not ubiquitous, and cannot get items by inspiration. Every dental society then, should open its doors, and either make its papers and proceedings public property for every recognized journal, or select some one of them as its medium of communication with the rest of the profession, and furnish it with a fair report of all that it has of interest for others. If however, any man or society desires to be left out in the gathering and distribution of professional knowledge, there is no law, save that of the common code of ethics, that will force him or them to

ARMY MEDICAL LIBRARY
WASHINGTON, D. C. U. S. A.

fall into line with the rest of the profession, though they are liable to the charge of deliberately courting the obscurity to which such an exclusive policy must surely doom them.

